# Novel genes and variants associated with congenital pituitary hormone deficiency in the era of next-generation sequencing

**DOI:** 10.3389/fendo.2022.1008306

**Published:** 2022-09-27

**Authors:** Hironori Bando, Shin Urai, Keitaro Kanie, Yuriko Sasaki, Masaaki Yamamoto, Hidenori Fukuoka, Genzo Iguchi, Sally A. Camper

**Affiliations:** ^1^ Division of Diabetes and Endocrinology, Department of Internal Medicine, Kobe University Hospital, Kobe, Japan; ^2^ Division of Diabetes and Endocrinology, Department of Internal Medicine, Kobe University School of Medicine, Kobe, Japan; ^3^ Division of Biosignal Pathophysiology, Kobe University Graduate School of Medicine, Kobe, Japan; ^4^ Medical Center for Student Health, Kobe University, Kobe, Japan; ^5^ Department of Human Genetics, University of Michigan, Ann Arbor, MI, United States

**Keywords:** combined pituitary hormone deficiency, hypopituitarism, next-generation sequencing, pituitary development, genetic diagnosis

## Abstract

Combined pituitary hormone deficiency (CPHD) is not a rare disorder, with a frequency of approximately 1 case per 4,000 live births. However, in most cases, a genetic diagnosis is not available. Furthermore, the diagnosis is challenging because no clear correlation exists between the pituitary hormones affected and the gene(s) responsible for the disorder. Next-generation sequencing (NGS) has recently been widely used to identify novel genes that cause (or putatively cause) CPHD. This review outlines causative genes for CPHD that have been newly reported in recent years. Moreover, novel variants of known CPHD-related genes (*POU1F1* and *GH1* genes) that contribute to CPHD through unique mechanisms are also discussed in this review. From a clinical perspective, variants in some of the recently identified causative genes result in extra-pituitary phenotypes. Clinical research on the related symptoms and basic research on pituitary formation may help in inferring the causative gene(s) of CPHD. Future NGS analysis of a large number of CPHD cases may reveal new genes related to pituitary development. Clarifying the causative genes of CPHD may help to understand the process of pituitary development. We hope that future innovations will lead to the identification of genes responsible for CPHD and pituitary development.

## Introduction

Combined pituitary hormone deficiency (CPHD) is defined as a deficiency of two or more pituitary hormones. The lack of hormones can affect the development of many parts of the body. Clinical presentation is variable, depending on the specific pituitary hormones that are deficient, although the most common symptoms include short stature, developmental delay, or delayed puberty.

CPHD is caused by variants in genes that regulate pituitary development (1). The incidence of CPHD is estimated at approximately 1 in 4,000 live births. However, most patients with CPHD (84%) have not been genetically diagnosed. CPHD is a multifactorial disorder that is usually associated with pituitary hypoplasia. Sanger sequencing of single genes identified in animal models has served as the conventional method for identifying genes and variants that cause the disease. Clearly, this method is limited, and it has been superseded in recent years by next generation sequencing (NGS) and chromosomal microarray analysis to detect copy-number variations.

Recent advances in the high-throughput analysis have exponentially advanced the pace of discovering novel variants associated with CPHD. Variants in genes previously implicated in isolated hypogonadotropic hypogonadism (IHH), septo-optic dysplasia (SOD), and holoprosencephaly (HPE) also cause CPHD. This illustrates that CPHD is part of a spectrum disorder, sometimes involving other craniofacial organs, such as the brain and eyes. Newly identified CPHD-associated genes provide clues to understanding new features of pituitary development. In addition, high-throughput analyses offer the opportunity to identify cases of oligogenic disease, in which variants in multiple genes collaborate to produce the clinical features.

We searched for articles related to CPHD that were not discussed in previously published review articles [such as (1)] and have summarized the recent discovery of new genes below. It can be difficult to ascertain the pathogenicity of genetic variants unless multiple, unrelated families with similar clinical features and lesions in the same gene are known, and/or convincing functional studies have been reported. We also present examples of known or suspected CPHD-associated genes that are supported by important confirmatory evidence. This review focuses on recent findings for novel genes that cause CPHD and on novel variants of known CPHD-related genes that drive CPHD through unique mechanisms. Most recent progress has been made by NGS analyses, such as exome, whole genome, and panel sequencing. Abnormalities in these genes generate characteristics typical of extra-pituitary phenotypes. From a clinical viewpoint, studying phenotypes other than pituitary function for the proper diagnosis and inference of causative genes seems necessary (**Table 1**).

**Table 1:** Characteristics of extra-pituitary abnormalities.

Gene	Associated symptoms	Reference(s) of the reported case
*B3GAT3*	Larsen-like syndrome (short stature, skeletal deformities, and congenital heart defects)	(2)
*BLM*	Bloom’s syndrome (predisposition to cancer, sun-sensitive skin rash, immune deficiency, and increased insulin resistance)	(3)
*FOXA2*	Hyperinsulinemia and biliary abnormalities	(4–6)
*L1CAM*	CRASH syndrome (corpus callosum hypoplasia, retardation, adducted thumbs, spasticity, and hydrocephalus)	(7)
*LAMB2*	Albuminuria due to congenital nephrosis and optical abnormalities	(8)
*MAGEL2*	Schaaf–Yang syndrome (hypotonia, feeding difficulties during infancy, global developmental delay, and sleep apnea)	(7)
*MIR17HG*	Feingold syndrome type 2 (microcephaly, learning disabilities, and digital anomalies)	(9)
*NKX2.1*	Brain–lung–thyroid syndrome (primary hypothyroidism, respiratory distress, and neurological disturbances)	(10, 11)
*RNPC3*	Delayed puberty, congenital cataracts, and developmental delay/intellectual deficiency	(12–14)
*ROBO1*	Ocular abnormalities, broad forehead, micrognathia, a broad philtrum, and arched eyebrows	(15–17)
*SEMA3A*	Heart, pelvic genitourinary dysplasia, and skeletal abnormalities	(18, 19)
*SMCHD1*	Bosma arhinia microphthalmia syndrome (microphthalmia and absence of a nose)	(20)

## Novel genes and variants identified by high-throughput analysis

### β-1,3-glucuronyltransferase 3 (B3GAT3)

GlcAT-I is one of the glucuronyltransferases that regulates the biosynthesis of glycosaminoglycan-protein linkers for proteoglycans (21). Proteoglycans are essential for cell–cell communications. *B3GAT3* gene encodes the GlcAT-I protein. Disruption of the linkage region due to mutations in *B3GAT3* has been reported to cause severe developmental defects. For example, homozygous *B3GAT3* variants have been associated with Larsen-like syndrome, which is characterized by short stature, skeletal deformities, and congenital heart defects (22). Bloor et al. reported a case where a patient had severe short stature, growth hormone (GH) deficiency, facial dysmorphisms, and congenital heart defects due to a heterozygous splice site mutation (c.888+262T>G) in the invariant “GT” splice donor site of *B3GAT3* gene (2).

The detailed mechanisms whereby *B3GAT3* variants cause GH deficiency have not been clarified.

### BLM recQ-like helicase (BLM)

*BLM* encodes a 3′−5′ ATP-dependent RecQ DNA helicase that plays an essential role in maintaining the genomic stability of DNA in somatic cells. Loss of BLM function causes chromosomal instability and increased sister-chromatid exchanges (23). BLM variants cause a rare autosomal-recessive genetic disorder named Bloom’s syndrome, which is characterized by short stature, predisposition to the development of cancer, sun-sensitive skin rash, immune deficiency, and increased risk for diabetes due to insulin resistance. GH deficiency was not thought to directly cause the short stature associated with this syndrome (24).

Verpula and colleagues reported a case of isolated growth hormone deficiency (IGHD) accompanied by facial photosensitive telangiectatic lesions, multiple café au lait spots, microcephaly, micrognathia, bilateral cryptorchidism, and recurrent systemic infections (3). The magnetic resonance imaging (MRI) findings showed a hypoplastic anterior pituitary gland and normal posterior pituitary gland. Genetic testing revealed a *BLM* variant (c.1489C>T, p.Gln497Ter). The causes of the hypoplastic anterior pituitary gland and GHD have not been reported.

### Activating mutations in B-Raf proto-oncogene, serine/threonine kinase (BRAF)

BRAF regulates the mitogen-activated protein kinase/extracellular signal-regulated kinase signaling pathway, which controls cell division, differentiation, and secretion. BRAF p.V600E is a well-known activating mutation that causes several types of tumors such as papillary craniopharyngioma, papillary thyroid carcinoma, colorectal cancer, melanoma, and non-small-cell lung cancer (25, 26). In addition, adrenocorticotropic hormone (ACTH)-producing pituitary adenoma and other activating variants have been reported.

Gualtieri and colleagues recently reported four activating mutations (p.Q257R, p.T241P, p.F468S, and p.G469E) in the *BRAF* gene in five patients with CPHD or SOD with cardio-facio-cutaneous syndrome (27). A mouse model of activated Braf-dependent anterior pituitary gland hypoplasia (*Prop1:Cre; Braf^V600E/+^*) exhibited dwarfism. These mice lacked GH, TSH, and LH and had increase of pro-opiomelanocortin (POMC) and prolactin (PRL) expression. Abnormal cell-lineage specification was associated with increase production of the lineage-specific transcription factor, T-box transcription factor 19, and decreased production of the transcription factor, POU class 1 homeobox 1 (POU1F1, also known as PIT-1). A proportion of SRY-box transcription factor (SOX) 2-positive progenitor/stem cells co-express POMC and PRL. These findings showed that BRAF plays a critical developmental role for the pituitary gland.

### Fibroblast growth factor receptor 1 (FGFR1)

FGFR1 is a receptor for fibroblast growth factor (FGF) 8, a signaling molecule important for pituitary gland formation. This receptor is mainly expressed in Rathke’s pouch and the ventral diencephalon during the embryonic period. FGFR1 variants cause isolated hypogonadotropic hypogonadism (IHH) or Kallmann syndrome (KS) *via* autosomal-dominant inheritance. FGFR1 variants cause seven to 10% of all cases of IHH or KS (28). Whole-exome sequencing was used to detect heterozygous nonsense variants in the FGFR1 gene (c.1864 C>T, p.R622X) in a sample from a patient with CPHD, delayed puberty, and micropenis (29). This variant was also reported for a familial case of IHH (30), suggesting that IHH caused by FGFR1 variants represents a milder phenotype of CPHD.

### Forkhead box A2 (FOXA2)

FOXA2 (also known as hepatocyte nuclear factor 3-beta, or HNF-3B) regulates the formation of ventral midline structures. Heterozygous deletions in the FOXA2 gene have been reported for patients with hypopituitarism and biliary abnormalities (4, 31). Recently, several *FOXA2* gene variants have been reported (c.505T>C [p.S169P] and c.770G>T [p.R257L], c.616C>T [p.Q206X]) for patients with hypopituitarism, thin pituitary stalks, and hypoplastic anterior pituitary glands (4–6). These symptoms were accompanied by hyperinsulinemia, which is caused by dysregulated insulin secretion due to reduced expression of ATP binding cassette subfamily C member 8 (ABCC8) and potassium inwardly rectifying channel subfamily J member 11 (KCNJ11). Variants in FOXA2 might serve as diagnostic markers for patients with hyperinsulinemia with hypopituitarism. A mouse study revealed that Foxa2 was expressed in the ventral hypothalamus and anterior pituitary gland (5). FOXA2 expression was accompanied by NK2 homeobox 2 (NKX2.2) expression, which shows interactions between the Shh/Gli signaling pathway; therefore, FOXA2 variants may cause pituitary hypoplasia.

### Immunoglobulin superfamily member (IGSF) 10

IGSF10 regulates the early migration of neurons expressing gonadotropin hormone-releasing hormone (GnRH) during embryogenesis (32). Variants of this gene cause self-limited delayed puberty or IHH because of dysregulated GnRH-induced neuronal migration. Budny and colleagues found a likely pathogenic variant (according to criteria from the American College of Medical Genetics and Genomics) of IGSF10 (c.5014G>A, p.A1672T) in patients with CPHD who lacked mutations in PROP paired-like homeobox 1 (PROP1) by performing whole-exome sequencing (18). Pituitary MRI showed the presence of a hypoplastic pituitary gland. The authors also reported two variants of IGSF10, although these variants were considered a variant of uncertain significance or a benign variant. Therefore, whether IGSF10 is actually involved in CPHD development requires further investigation.

### L1 cell-adhesion molecule (L1CAM)

L1CAM is a cell-adhesion molecule of the immunoglobulin superfamily that regulates neuronal cell adhesion, migration, myelination, and neuronal differentiation (33). Variants of the *L1CAM* gene cause L1 syndrome (also known as a CRASH syndrome), which is characterized by corpus callosum hypoplasia, retardation (intellectual disability), adducted thumbs, spasticity, and hydrocephalus (34). An *L1CAM* variant (c.1354G.A, p.G452R) was detected in a patient with GHD and systemic abnormalities, such as clubbed hands, plagiocephaly, global developmental delay, hypotonia, arthrogryposis, divergent squint, and hydrocephalus (7). The patient’s brain was underdeveloped with a very thin corpus callosum. The pathogenesis of the pituitary abnormality has not been clarified, but *L1CAM* expression was detected in the hypothalamus, not in Rathke’s pouch (7). Decreased signaling related to pituitary gland formation by the hypoplastic hypothalamus may have caused the hypopituitarism.

### Laminin subunit beta 2 (LAMB2)

Laminin b2 is abundantly expressed in the glomerular basement membrane. LAMB2 variants cause congenital nephrosis with mesangial sclerosis and optical abnormalities (35). In 2020, compound heterozygous missense mutations identified in *LAMB2* (c.737G>A [p.Arg246Gln] and c.3982G>C [p.Gly1328Arg]) were detected in a patient with isolated GH deficiency and global developmental delay, hypoplastic anterior pituitary, optic nerve hypoplasia, and corpus callosum dysgenesis (8). The details have not been investigated, but the pituitary glands of *Lamb2* ^–/–^ mice were found to have abnormal cellular clusters. LAMB2 is expressed in the epithelium of Rathke’s pouch, suggesting that LAMB2 has an essential role in forming Rathke’s pouch (36). From a clinical perspective, the observation of albuminuria may help identify LAMB2 variants in patients with SOD.

### MAGE family member L2 (MAGEL2)

*MAGEL2*, a maternally imprinted gene, is one gene contained within the Prader–Willi locus. *MAGEL2* variants cause Schaaf–Yang syndrome, which is characterized by symptoms that resemble Prader–Willi syndrome, such as hypotonia, feeding difficulties during infancy, global developmental delay, and sleep apnea (but lack certain stereotypical Prader–Willi syndrome features, such as hyperphagia and subsequent obesity). *Magel2*-null mice showed a phenotype including neonatal growth retardation, excessive weight gain, impaired hypothalamic regulation, and infertility (37–39). A heterozygotes mutation in the *MAGEL2* gene (c.1996dupC, p.Q666Pfs*47) was reported for patients with Schaaf–Yang syndrome accompanied by CPHD (some cases showed complications with central diabetes insipidus) (7). The MRI findings of the anterior and posterior pituitary glands were variable.

### Microdeletions of chromosome 13q31.3, including the miR-17-92a-1 cluster host gene (*MIR17HG*)

Feingold syndrome type 2 (FS2) is a rare genetic congenital-malformation syndrome that is characterized by microcephaly, learning disabilities, short stature, and digital anomalies (brachymesophalangy, fifth finger clinodactyly, syndactyly of toes, and hypoplastic thumbs) (40). Deletions of chromosome 13q31.3, including the *MIR17HG* gene, have been implicated as the cause of FS2.

A patient with a cardiac anomaly, gastroesophageal reflux disease, global developmental delay, hypotonia, and developmental dysplasia showed growth deficiency with an empty sella and a normal neurohypophysis (9). A single-nucleotide polymorphism-based microarray revealed an ~8 Mb deletion at 13q31.3q32.3, including the *MIR17HG* gene that causes FS2.

The causes of GH deficiency have not been determined. Recent findings revealed that several microRNAs regulate pituitary gland development (41). *MIR17HG* might regulate pituitary development or hormone secretion. However, the deletions in chromosome 13q31.3 also contain *SOX21*. *Sox21* deletion caused a postnatal growth deficiency due to increased energy expenditure without obvious pituitary abnormalities (42). Therefore, we have to be aware of the association of the *SOX21* deletion in patients with FS2 and a short stature.

### NK2 homeobox 1 (NKX2.1)

NKX2-1 (also known as TTF-1) is a transcription factor that regulates organogenesis and differentiation of the thyroid gland, lungs, and ventral forebrain, including the hypothalamus. *NKX2.1* variants cause primary hypothyroidism, respiratory distress, and neurological disturbances (brain–lung–thyroid syndrome). Several patients with *NKX2.1* variants have been found to develop hypothalamic disorders such as temperature dysregulation and dysrhythmic sleep; however, CPHD has not been associated with *NKX2.1* variants. A familial case with motor-development delay, mixed-movement disorder, and endocrinological abnormalities (father: hypogonadotropic hypogonadism, daughter: GH deficiency) had a pathogenic stop variation in *NKX2.1* (c.338G>A, p.Trp113∗) (10). In addition, a patient with deletion containing the *NKX2.1* gene had CPHD (GH, ACTH, TSH, and gonadotropin) with a small anterior pituitary gland, but without optic nerve hypoplasia (11). *Nkx2-1* is expressed in the developing ventral diencephalon, and a mouse model showed that the absence of this gene caused defects in both Rathke’s pouch and ventral diencephalon development (43). The expression of *Fgf8*, a potent inducer of Rathke’s pouch growth, was not detectable in the ventral diencephalon of *Nkx2.1* null embryos.

### RNA-binding region (RNP1, RRM)-containing 3 (RNPC3)

The *RNPC3* gene encodes the U11/U12-65K protein, a component of the minor spliceosome. The minor spliceosome catalyzes the removal of U12-type spliceosomal introns from eukaryotic messenger RNAs (44, 45). This minor spliceosome regulates 0.35% of all human introns (46).

Recently, several groups reported variants of the *RNPC3* gene in patients with CPHD (GH [inevitable], PRL [undetectable-normal], and TSH [occasional] deficiencies), as determined by NGS (12–14). These variants showed various phenotypes, such as delayed puberty, congenital cataracts, and developmental delay/intellectual deficiency. The MRI findings of the anterior pituitary gland were hypoplastic to normal.

Several pituitary hormone-related genes whose introns are regulated by RNPC3 have been implicated, but the associated regulatory mechanisms are unclear (13).

### Roundabout guidance receptor 1 (ROBO1)

v*ROBO1* is a member of the immunoglobulin gene superfamily and encodes an integral membrane protein. This protein is a receptor for Slit homolog (Slit) proteins and serves an essential role in axon guidance and neuronal precursor cell migration in the forebrain (47). *Robo1*-knockout mice have an embryonic-lethal phenotype. These embryos showed dysgenesis of the corpus callosum, hippocampal commissure, and corticothalamic and thalamocortical targeting abnormalities.

Recently, Bashamboo et al. reported the first case of CPHD accompanied by pituitary stalk interruption syndrome (PSIS) (15). Subsequently, several groups reported variants of the *ROBO1* gene in patients with similar pituitary and pituitary stalk phenotypes (15–17). All patients showed pituitary hypoplasia both in the posterior pituitary (PSIS or invisible stalk) and the anterior pituitary (small or absent). Most patients with *ROBO1* variants also showed craniofacial phenotypes, including ocular abnormalities (i.e., hypermetropia with strabismus and ptosis), a broad forehead, micrognathia, a broad philtrum, and arched eyebrows.

The mechanisms of Slit/ROBO1 in the development of the pituitary stalk and anterior pituitary have not been clarified. However, regulation of neurogenic locus notch homolog protein (Notch)/hairy and enhancer of split-1 (Hes1) signaling by ROBO1 was suspected because *Hes1* plays a specific role in guiding hypothalamic axons to the pituitary gland (48).

### Semaphorin 3A (SEMA3A)

Neurons and surrounding tissue secrete SEMA3A to guide migrating cells and axons in the developing nervous system, including the hypothalamus. Heterozygous variants of this gene cause IHH and KS (49, 50). A patient with short stature and multiple anomalies (such as macrocephaly, thoracic bone skeleton, heart defect, and camptodactyly) due to a heterozygous 150 kb deletion, including part of the *SEMA3A* gene, was reported (51). However, that case report did not discuss of GH or pituitary hormones.

Recently, whole-exome sequencing detected a likely pathogenic variant of SEMA3A (c.1302_1303delinsCA, p.V435delinsI) in a patient with CPHD accompanied by PSIS (18). Another variant (c.950A>G) of this gene was found in a patient with CPHD accompanied by multiple abnormalities (heart, pelvic genitourinary dysplasia, and skeletal) (19). The pituitary MRI showed a hypoplastic pituitary gland.

An association of SEMA3A with pituitary development has not been reported, but *Sema3a* was detected in the ventral diencephalon and oral ectoderm of E10.5 mouse embryos (Genepaint, https://gp3.mpg.de/results/(semaphorin)%203A (52); therefore, this gene might be involved in pituitary development.

### Structural maintenance of chromosomes flexible hinge domain-containing 1 (SMCHD1)

SMCHD1 regulates DNA methylation of multiple genomic loci and can result in X-chromosome inactivation (53). Recent findings revealed that *SMCHD1* is maternally imprinted (54). *SMCHD1* variants are causative for Bosma arhinia microphthalmia syndrome, which is characterized by the absence of a nose, microphthalmia, and IHH. Kinjo and colleagues screened for *SMCHD1* gene variants in 43 patients with CPHD with normal noses and no pathogenic variants in any known causative genes. The authors identified an *SMCHD1* variant (c.G1192A [p.Asp398Asn]) in a patient with CPHD (GH, ACTH, TSH, and gonadotropin deficiency) accompanied by mild intellectual disability (20). The anterior pituitary lobe was missing and the patient had ectopic posterior pituitary. The structures of the eyes and nose were normal, but the optic nerve was hypoplastic.

## Recent insights into synonymous variants and abnormal RNA processing in CPHD

Synonymous variants (also called ‘silent’ mutations) are now widely known to cause changes in protein expression levels, conformations, and functions (55). Several diseases in most organ systems have been associated with synonymous mutations. Recent data revealed several molecular mechanisms underlying the changes in protein levels or confirmations due to synonymous mutations; 1) truncated mRNAs due to exon skipping that leads to alternative splicing, 2) mRNA-stability changes resulting in low protein levels, 3) decreased rates of protein synthesis leading to protein misfolding, and 4) “pause sites” that can result in alternative conformers during co-translational folding.

Alternative splicing may lead to a loss or alteration of a protein’s normal function, and alternative-splicing defects cause human diseases. For example, mutations in the intronic sequences of the ATPase copper transporting alpha (*ATP7A*) gene cause Menkes disease and occipital horn syndrome (56). A patient with Peutz–Jeghers syndrome was also reported to harbor a mutation in the intronic sequence of the liver kinase B1 (*LKB1*) gene (57). These mutations causes alternative-splicing defects and led to the translation of proteins with abnormal functions. In addition, recent data revealed that abnormal RNA processing by synonymous variants in pituitary-related genes caused CPHD.

POU1F1 (also known as PIT-1) plays essential roles in differentiating somatotrophs, lactotrophs, and thyrotrophs in the anterior pituitary gland. It also regulates the expression of GH, PRL, and TSH. Thus, *POU1F1* gene variants can cause CPHD through GH, PRL, and TSH deficiencies. *POU1F1* is expressed as two splice isoforms (the α- and β-isoforms). The difference between both isoforms is a 26-amino acid (78-base pair) in-frame insertion in the second exon, caused by alternative splice-acceptor utilization. The expression level of β-isoform is very low in human pituitaries (~1%), and this isoform suppresses GH, PRL, TSHβ, and POU1F1 promoter activity. Recently, two groups independently reported interesting POU1F1 variants, namely c.148T>G (p.Ser50Ala), c.150T>G (p.Ser50=), c.153T>A (p.Ile51=), c.152T>G (p.Ile51Ser), c.155T>G (p.Leu52Trp), and c.157T>G (p.Ser53Ala) at about the same time (58, 59). Their results suggested that β-domain variants in *POU1F1* cause pituitary deficiency due to dominant β-isoform expression. A high-throughput splicing-reporter assay revealed that 96 splice-disruptive variants (including 14 synonymous variants) out of 1,070 single-nucleotide variants in *POU1F1* affected alternative splicing.

Type-II IGHD is another example of CPHD that arises from alternative splicing (60). The pathogenesis of this disease involves single-base mutations within the first six nucleotides of intron 3, which affect abnormal growth hormone 1 (*GH1*) splicing (61). This abnormal exon skipping leads to the production of a 17.5 kDa isoform, which exerts a dominant-negative effect on the secretion of the bioactive 22 kDa isoform (62, 63). Accumulation of the 17.5 kDa isoform promotes endoplasmic reticulum stress in pituitary hormone-producing cells and causes decreased secretion of GH and multiple pituitary hormones (62).

Currently, CPHD has been reported to be caused by abnormal exon skipping in 2 genes, namely *POU1F1* and *GH1*. In the future, additional genes that cause CPHD by similar mechanisms may be identified.

## Conclusion

IHH, SOD, IPHD, and CPHD are on the same disease spectrum. In the future, we expect that additional genes will be identified that contribute to the development of these diseases. High-throughput analysis may help identify the causative gene(s). Previously, 30 genes associated with CPHS and 37 candidate genes were sequenced, but this method only identified the causative gene for CPHD in one out of 51 cases (64). Many more unknown causative genes likely exist. However, it is challenging to predict the phenotype from a genotype as environmental factors and oligogenic disease are likely contributors. If NGS were performed on a larger number of CPHD cases, it could lead to the identification of causative genes. The possibility cannot be ruled out that monogenetic diseases and oligogenic abnormalities may be associated with the disease (65). Moreover, the results of a recent study revealed copy-number variants in several genes that might contribute to the formation of CPHD (66).

Clarifying the causative genes of CPHD might rewrite our understanding of the process of pituitary development. In addition, basic research on pituitary formation may aid in inferring causative genes for CPHD.

## Author contributions

Writing draft, HB and GI. Searching newly reported genes, HB, SU, KK, YS, MY, and HF. Supervision of the article, SC. All authors contributed to the article and approved the submitted version.

## Funding

This work was supported by Grants-in-Aid for Scientific Research from the Japan Society for the Promotion of Science (KAKENHI, grant numbers: 21K16370 and 21KK0149 to HB, 21K20933 to KK, and 21K08555 to GI), the Takeda Science Foundation (a medical research grant to HB), and the National Institutes of Health (grant number R01HD097096 to SC).

## Acknowledgments

The authors thank our laboratory members for their fruitful discussions and suggestions.

## Conflict of interest

HB has received research grants from Bristol Myers Squibb.

The remaining authors declare that the research was conducted in the absence of any commercial or financial relationships that could be construed as a potential conflict of interest.

## Publisher’s note

All claims expressed in this article are solely those of the authors and do not necessarily represent those of their affiliated organizations, or those of the publisher, the editors and the reviewers. Any product that may be evaluated in this article, or claim that may be made by its manufacturer, is not guaranteed or endorsed by the publisher.

## Glossary

**Table:**

ABCC8	ATP binding cassette subfamily C member 8
ACTH	Adrenocorticotropic hormone
ATP7A	ATPase copper transporting alpha
B3GAT3	β-1, 3-glucuronyltransferase 3
BLM	BLM RecQ-like helicase
BRAF	B-Raf proto-oncogene, serine/threonine kinase
CPHD	combined pituitary hormone deficiency
FGF	fibroblast growth factor
FGFR1	fibroblast growth factor receptor 1
FOXA2	forkhead box A2
FS2	Feingold syndrome type 2
GH	growth hormone
GH1	growth hormone 1
GnRH	gonadotropin hormone-releasing hormone
Hes1	hairy and enhancer of split-1
HPE	holoprosencephaly
IGHD	isolated growth hormone deficiency
IHH	isolated hypogonadotropic hypogonadism
ISGF	immunoglobulin superfamily member
KCNJ11	potassium inwardly rectifying channel subfamily J member 11
KS	Kallmann syndrome
L1CAM	L1 cell-adhesion molecule
LAMB2	laminin subunit beta 2
LH	luteinizing hormone
LKB1	liver kinase B1
MAGEL2	MAGE family member L2
MIR17HG	miR-17-92a-1 cluster host gene
NGS	next-generation sequencing
NKX2.1	NK2 homeobox 1
NKX2.2	NK2 homeobox 2
Notch	neurogenic locus notch homolog protein
POMC	pro-opiomelanocortin
POU1F1	POU class 1 homeobox 1
PRL	prolactin
PROP1	PROP paired-like homeobox 1
PSIS	pituitary stalk interruption syndrome
RNPC3	RNA-binding region (RNP1, RRM)-containing 3
ROBO1	roundabout guidance receptor 1
SEMA3A	semaphorin 3A
Slit	Slit homolog
SMCHD1	structural maintenance of chromosomes flexible hinge domain-containing 1
SOD	septo-optic dysplasia
SOX	SRY-box transcription factor
TSH	thyroid-stimulating hormone
